# Screening for vitamin D deficiency in a tropical area: results of a sun exposure questionnaire

**DOI:** 10.1186/s12902-018-0272-0

**Published:** 2018-07-03

**Authors:** Fernanda Barros Bittar, Charlles H. M. Castro, Vera Lúcia Szejnfeld

**Affiliations:** Rheumatology Division, Universidade Federal de São Paulo - Escola Paulista de Medicina (UNIFESP-EPM), Rua Botucatu, 740 – 3° andar – Vila Clementino, CEP, São Paulo, SP 04023-900 Brazil

**Keywords:** Questionnaire, Sun exposure, Vitamin D, Young, And elderly, Summer, Winter

## Abstract

**Background:**

Vitamin D deficiency is pandemic while resources available to measure 25-hydroxyvitamin D (25OHD) are limited. The present study aimed to verify whether sun exposure measured by a structured questionnaire could predict serum 25OHD concentrations in healthy Caucasian individuals living in a tropical area.

**Methods:**

A cross-sectional study was carried out in subjects living in the greater São Paulo area, Brazil. Two groups of 50 young (20 to 40 years old) and 50 older (60 to 80 years old) subjects (*N* = 200) answered a structured questionnaire on sun exposure and had blood samples drawn for serum 25OHD concentration measurement during both summer and winter. Anthropometric data were also recorded. Correlation between the questionnaire variables (duration of sun exposure, amount of exposed skin, total sun exposure score - TSES and other data) and serum 25OHD concentration was evaluated.

**Results:**

Mean serum 25OHD concentration was 17.60 ± 7.3 ng/mL with no difference between age groups (*p* = 0.293). TSES weakly correlated with serum 25OHD levels (*r* = 0.264; *p* < 0.001). Separate analyzes by age groups demonstrated that TSES correlated significantly with serum 25OHD concentration only in the older subjects during summer (*r* = 0.322; *p* = 0.023). Using linear regression analyses, TSES and body mass index (BMI) were significantly associated with serum 25OHD levels. On the other hand, Receiver operating characteristic (ROC) analysis for TSES showed no significance as a screening tool for vitamin D deficiency (*p* = 0.172).

**Conclusion:**

Sun exposure questionnaire associated with BMI correlates with serum 25OHD concentration with very low accuracy. The use of the questionnaire does not discriminate between vitamin D sufficient and deficient individuals.

**Electronic supplementary material:**

The online version of this article (10.1186/s12902-018-0272-0) contains supplementary material, which is available to authorized users.

## Background

Vitamin D is important for calcium and phosphorus homeostasis, with a fundamental participation in bone mineralization and remodeling [1]. It has been demonstrated that vitamin D plays an important role in bone development in children, maintenance of bone in adults and prevention of osteoporosis in the elderly [2, 3]. In humans the main source for vitamin D comes from the conversion of 7-dehydrocholesterol precursors in the skin through interaction with ultraviolet B (UVB) radiation. 7-dehydrocholesterol UVB-induced conversion to cholecalciferol is dependent on latitude, time of the day, season, duration of exposure and amount of exposed skin, as well as age and cutaneous pigmentation [4]. Vitamin D synthesis in the skin under UVB radiation is significantly limited at latitudes > 33°, including North America and Europe. In those locations the sun is only an effective source of vitamin D during the summertime [5]. Dietary sources of vitamin D are limited and predominantly comprise fatty fish and nuts [6].

Vitamin D deficiency is currently one of the most common and neglected medical conditions, affecting a large part of the world population [1, 7]. The increasing concern about the high prevalence of vitamin D deficiency and its skeletal consequences especially among the elderly has led to a large increase in the demand for serum 25OHD measurements, currently the best marker of vitamin D status [8]. Simple and low-cost methods that allow for direct measurement of 25OHD in patients who are at risk of deficiency are needed [9, 10]. The economical impact of such strategies would be rather strong among developing countries with no solid initiatives for public health care [11].

We have recently reported a very high prevalence of vitamin D deficiency among Brazilian men and women [12]. Vitamin D deficiency is pandemic and can be observed even in a sunny country like Brazil where excessive sunlight exposure related outcomes such as non-melanoma skin cancer are highly prevalent [13, 14].

The variables that may affect 25OHD status are relatively well defined [4, 7]. On the other hand, only a few studies have designed indirect tools to predict serum 25OHD concentrations and identify individuals who may be at risk of vitamin D deficiency [15]. The use of specific tools to rationally indicate 25OHD testing would be welcome in our current scenario where extensive screening for vitamin D deficiency is not recommended [16]. In the present study we hypothesize that sun exposure assessment by a structured questionnaire [17] may correlate with serum 25OHD measurement and thus could be helpful as an indicator for 25OHD testing in adults with suspected vitamin D deficiency.

## Methods

### Participants

A total of 200 healthy Caucasian men and women living in the greater São Paulo area, Brazil, were invited to participate. Participants were divided into 4 groups: 50 young participants aged 20 to 40 years old during summer, 50 young participants during winter, 50 older participants aged 60 to 80 years old during summer and 50 older participants during winter. All participants were Caucasian with Fitzpatrick skin phototype I to III [18]. Exclusion criteria were: Non-Caucasian individuals and Fitzpatrick skin phototype IV and higher, age outside the study bands, conditions that could impair 25OHD metabolism and use of vitamin D supplementation. Vitamin D supplements were defined as all oral supplements containing vitamin D other than food. The use of sunscreen was also an exclusion criterion. Sunscreen use was defined as the use of any sunscreen products within 3 months prior to the study regardless of the frequency. Participants were volunteers and gave written informed consent before entering the study. UNIFESP/EPM’s Ethics and Research Committee approved the present study (reference number 464.939).

### Data collection

The study was conducted between December 2013 and February 2014 (summer in the south hemisphere) and between June 2014 and August 2014 (winter). Blood samples were collected in photoprotected tubes and stored at − 70 °C until serum 25OHD was measured. Serum 25OHD measurements were performed using chemiluminescence technique with a coefficient of variation of 11.7% [19]. Vitamin D status was classified as normal when serum 25OHD concentration was ≥20 ng/mL and deficient when < 20 ng/mL, according to the recommendation from the American Institute of Medicine (IOM) [6]. The Endocrine Society definition criteria for vitamin D deficiency were also evaluated [9].

Simultaneously with blood collection all the participants answered a questionnaire on daily sun exposure correspondent to the previous week, as formerly published [17]. Sun exposure between 9:00 AM and 4:00 PM was recorded. Scores in the questionnaire are based on the duration of sun exposure (Time in sun: less than 5 min = 0, between 5 and 30 min = 1 and greater than 30 min = 2) and in the amount of exposed skin surface (Skin Exposure: face and hands = 1; face, hands and arms = 2; face, hands and legs = 3; bathing suit = 4). Scores were calculated for each day of the previous week. A final score to estimate the mean amount of sun exposure was calculated as the product of the amount of time by the amount of exposed skin. This final score was calculated for each day to reflect the Daily Sun Exposure Score (minimum = 0, maximum = 8). All seven Daily Sun Exposure Scores were summed to generate the Total Sun Exposure Score (TSES) for the entire week (minimum = 0, maximum = 56). We also considered the Weekly Time of Exposure Score and the Weekly Skin Amount Score, which were calculated by adding the scores corresponding to each day of the week (minimum = 0 and maximum = 14; minimum = 7 and maximum = 28, respectively). Body weight was measured (after removal of shoes and heavy outer clothing) using a balance beam scale. Height was measured (after removal of shoes) using a Filizola® stadiometer. Height and weight were used to calculate the body mass index (BMI; kg/m^2^).

### Statistical analyzes

All statistical analysis was performed using SPSS 19.0 software package (SPSS Inc., Chicago, IL). Mann-Whitney test was used to analyze the anthropometric data between independent groups. Student’s *t*-test was used to compare TSES as well as mean serum 25OHD concentrations between groups. A linear regression model was used to identify variables associated with serum 25OHD measurements while logistic regression models were used to identify anthropometric and questionnaire variables that were associated with vitamin D deficiency. Receiver operating characteristic (ROC) curve analysis was performed to determine the performance of TSES to predict vitamin D deficiency (serum 25OHD concentration below 20 ng/mL). Significance level was set as *p* < 0.05.

## Results

Table 1 shows anthropometric data and vitamin D status for the study participants. Using the IOM definition criteria [6] we observed a very high prevalence of vitamin D deficiency. A total of 133 subjects (66.5%) had serum 25OHD concentration below 20 ng/mL. Mean serum 25OHD concentrations did not differ significantly between young and old subjects (18.27 ± 7.13 ng/mL and 16.93 ± 7.45 ng/mL, respectively, *p* = 0.29). Significant effect of seasonality on 25OHD measurement was detected in young participants. For this age group mean serum 25OHD concentration was significantly higher in the summer than that measured in the winter (21.58 ± 6.08 ng/mL and 14.95 ± 6.58 ng/mL, respectively, *p* = 0.003). The same was not observed in the older participants.

**Table 1:** Anthropometric data and serum 25-hydroxyvitamin D concentration in young (20 to 40 years old) and old (60 to 80 years old) healthy Caucasian individuals living in São Paulo, Brazil

	All (*N* = 200)	Young (*N* = 100)	Old (*N* = 100)	*p* ^a^
Age (years, mean ± SD)	49.6 ± 19.4	31.5 ± 7.0	67.8 ± 6.4	–
Sex (Men: Women, N)	52: 148	19: 81	33: 67	0.02
BMI (kg/m^2^, mean ± SD)	26.44 ± 4.76	25.85 ± 3.57	27.04 ± 4.62	0.05
25OHD (ng/mL, mean ± SD)	17.6 ± 7.3	18.27 ± 7.13	16.93 ± 7.45	0.29
25OHD < 20 ng/mL (N, %)	133 (66.5%)	66 (66%)	67 (67%)	0.36

*25OHD* 25-hydroxyvitamin D, *BMI* body mass index

^a^Chi-squared or Mann-Whitney

The overall mean TSES was 14.9 ± 9.58 (maximum was 28). Young participants had TSES significantly higher than those observed for the older group (*p* < 0.001). As expected, TSES were higher in the summer as compared to the winter in both age groups (*p* = 0.005).

**Fig. 1:**
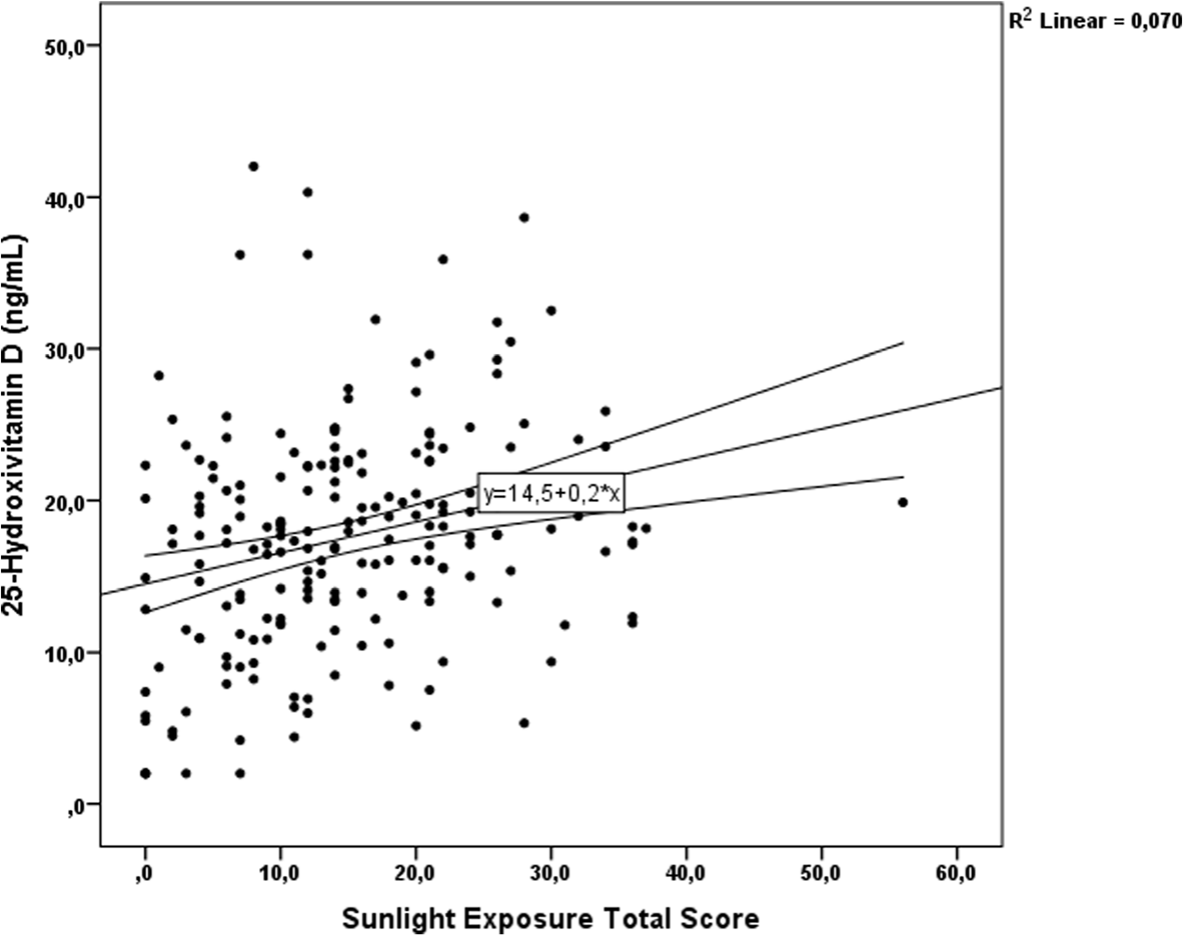
Linear regression between serum 25-hydroxyvitamin D concentrations and total sun exposure score in 200 healthy Caucasian Brazilian individuals

We observed modest although statistically significant correlation between TSES and serum 25OHD concentration (*r* = 0.264, p < 0.001), as shown in Fig. 1. This association was particularly confirmed when the older participants were analyzed separately. In this age group, a weak but significant correlation was found between TSES, Weekly Time of Exposure Score and serum 25OHD concentrations only in the summer (*r* = 0.322, *p* = 0.023 and *r* = 0.433, *p* = 0.002, respectively). For the young age group, none of the variables of the questionnaire correlated significantly with serum 25OHD concentration neither in the summer nor in the winter.

Multiple linear regression analyses demonstrated that TSES and BMI were significantly associated with serum 25OHD measurements, as shown in Table 2. The equation was significant (*R* = 0.297), however the analyzed variables explained only 8.8% of the variability of serum 25OHD measurements (R^2^ = 0.088).

**Table 2:** Multiple linear regression analyses for serum 25-hydroxyvitamin D concentration in healthy Caucasian Brazilian individuals living in São Paulo, Brazil

	Β (95% confidence interval)	Standard error	*p*
25OHD	20.052 [14.238/25.866]	2.948	< 0.001
TSES	0.206 [0.102/0.310]	0.053	< 0.001
BMI	- 0.211 [−0.420/− 0.001]	0.106	0.048

*25OHD* 25-hydroxyvitamin D, *TSES* total sun exposure score, *BMI* body mass index

Using logistic regression analysis, an inverse correlation was found between BMI and 25OHD status (OR = 0.926, *p* = 0.027). Multiple logistic regression analysis demonstrated that BMI and TSES were the main variables associated with vitamin D deficiency (serum 25OHD below 20 ng/mL), as shown in Table 2. The performance of TSES to predict vitamin D deficiency (25OHD < 20 ng/mL) was considered poor with a non-significant area under the curve (AUC) (*p* = 0.172) (Additional file 1: Figure S1). Similar results were found when vitamin D deficiency was defined according to the Endocrine Society cut off value (25OHD < 30 ng/mL) [9].

## Discussion

Our results demonstrated that the sun exposure questionnaire proposed by Hanwell et al. [17] was not able to discriminate vitamin D sufficient from deficient individuals when applied to healthy young and old Caucasian subjects living in a tropical area in Brazil. TSES estimated by the questionnaire showed a significant but weak correlation with serum 25OHD concentration. The questionnaire thus did not hold as a reliable tool to guide or indicate serum 25OHD concentration measurement. Separate analyses by age group (young and older) or season (summer and winter) did not hold significance. As expected, TSES and BMI were significantly associated with serum 25OHD concentration but were able to explain just a minimal amount of its variability.

In Brazil, 99.3% of 2040 subjects evaluated in the population-based BRAZOS study had vitamin D intake below recommendation [7]. Likewise, a high prevalence of vitamin D deficiency has been systematically observed in several samples of our population [8–11]. The prevalence of vitamin D deficiency depends upon the definition criteria used [6, 9]. In our present study mean serum 25OHD measurements in the subgroups evaluated (with the exception of young subjects measured during winter) were greater than 16 ng/mL (with the exception of wintertime in young patients), level that covers the requirements of approximately half the population, according to the IOM [6].

The questionnaire we used in the present study is very easy to be applied and has shown satisfactory results in another Caucasian population with strong correlation with serum 25OHD levels (Spearman’s rho = 0.59) [17]. On the other hand, there was no significant association between TSES and serum 25OHD levels in a Canadian cohort [20]. Mean TSES in our sample (14.9) are low and could underscore a current trend of an indoor lifestyle. On the other hand, the moderate to low sun exposure measured by the questionnaire is not in agreement with the evidence about sun exposure in Brazil. Due to our tropical and subtropical climate, geographic characteristics and a culturally low frequency of photo protection, Brazilians are among the populations with the highest annual sun exposure [13]. The lack of association in our study and in the Canadian cohort [20] between sun exposure measured by the questionnaire and serum 25OHD concentration may, at least in part, reflect the poor accuracy of the questionnaire to measure sun exposure. Based on subjects’ recall of sun exposure in the previous week, the questionnaire may lack internal validity. The high level of atmospheric pollution in the greater São Paulo area [21] might have affected our current results since it reduces the ability of UVB radiation to induce vitamin D synthesis [22]. As expected, mean sun exposure was higher among young people as opposed to the elderly and also in the summer as compared to the winter.

It could sound unrealistic to hypothesize that a sun exposure questionnaire would give us an insight into the circulating 25OHD concentrations. Multiple reasons would certainly work against that hypothesis including time of day of exposure, season, skin phototype and area of sun exposure, use of sunscreen and other barrier factors. The study tried to take that into account and only recruited Caucasian individuals with light phototypes (I to III). Skin area of exposure was also calculated. The fact that sun exposure was recorded from 9 AM up to 4 PM can have a dramatic effect on the cutaneous synthesis of vitamin D: very early (between 9 and 10 AM) and late hours (3 to 4 PM) certainly are associated with significantly less vitamin D synthesis when compared to peak hours from 11 AM to 1 PM. Unfortunately the questionnaire used does not consider those observations and that might have had an effect on our results.

It has been now well established that obesity can contribute to hypovitaminosis D. Significant inverse correlation between BMI and serum 25OHD concentration seen in our sample has been described [15, 23, 24]. It is estimated that for every 10% increase in BMI there will be a 4.2% decrease in serum 25OHD concentration [23]. About 57% of our participants had BMI above 25 kg/m^2^ and 14% above 30 kg/m^2^.

Important limitations of the present study need to be pointed out. As other variables such as photo protection methods other than sunscreen, diet and physical activity were not considered, it could be inferred that such information could have also affected the relationship between the questionnaire variables and serum 25OHD concentration. More complex questionnaires including a number of other variables might have improved performance to identify individual at risk of vitamin D deficiency [25]. The questionnaire was tested in healthy Caucasian subjects with no clear recommendation for screening for vitamin D deficiency. Even though the prevalence of vitamin D deficiency in the study population was significantly high, using the questionnaire in patients with osteoporosis or at a higher risk for vitamin D deficiency might lead to different results. The cross sectional nature of the study design precludes us from establishing a cause effect relationship between the variables in analysis. Continuous follow-up of the same individuals at two seasons could reduce the differences depending on the individual characteristics, minimizing the variability of the data.

## Conclusion

In conclusion, in spite of being simple to use, the studied sun exposure questionnaire has very low accuracy to estimate serum 25OHD concentration. The questionnaire does not allow discriminating between vitamin D sufficient and deficient individuals in a tropical area. There remains the need of reliable tools to better indicate 25OHD testing and reduce costs of diagnosis and management of vitamin D deficiency.

## Additional file

Additional file 1: **Figure S1.** Receiver operating characteristic (ROC) curve showing the performance (sensitivity and specificity) of total sun exposure score to predict vitamin D deficiency (serum 25OHD concentration below 20 ng/mL) in healthy Caucasian individuals living in São Paulo, Brazil. Area under the curve = 0.559; *p* = 0.172. (DOCX 46 kb)

## Abbreviations

25OHD: 25-hydroxyvitamin D
AUC: Area under the curve
BMI: Body mass index
IOM: Institute of Medicine
ROC: Receiver operating characteristic
TSES: Total sun exposure score
UVB: Ultraviolet B

## References

[1] Holick MF (2017). The vitamin D deficiency pandemic: approaches for diagnosis, treatment and prevention. Rev Endocr Metab Disord.

[2] Abrahamsen B, Harvey NC (2013). The role of vitamin D supplementation in patients with rheumatic diseases. Nat Rev Rheumatol.

[3] Christakos S, DeLuca HF (2011). Minireview: vitamin D: is there a role in extraskeletal health?. Endocrinology.

[4] Holick MF (1995). Environmental factors that influence the cutaneous production of vitamin D. Am J Clin Nutr.

[5] Brouwer-Brolsma EM, Bischoff-Ferrari HA, Bouillon R, Feskens EJ, Gallagher CJ, Hypponen E, Llewellyn DJ, Stoecklin E, Dierkes J, Kies AK (2013). Vitamin D: do we get enough? A discussion between vitamin D experts in order to make a step towards the harmonisation of dietary reference intakes for vitamin D across Europe. Osteoporos Int.

[6] Ross AC, Manson JE, Abrams SA, Aloia JF, Brannon PM, Clinton SK, Durazo-Arvizu RA, Gallagher JC, Gallo RL, Jones G (2011). The 2011 report on dietary reference intakes for calcium and vitamin D from the Institute of Medicine: what clinicians need to know. J Clin Endocrinol Metab.

[7] Holick MF (2007). Vitamin D deficiency. N Engl J Med.

[8] DeLuca HF (2004). Overview of general physiologic features and functions of vitamin D. Am J Clin Nutr.

[9] Holick MF, Binkley NC, Bischoff-Ferrari HA, Gordon CM, Hanley DA, Heaney RP, Murad MH, Weaver CM (2011). Evaluation, treatment, and prevention of vitamin D deficiency: an Endocrine Society clinical practice guideline. J Clin Endocrinol Metab.

[10] Maeda SS, Borba VZ, Camargo MB, Silva DM, Borges JL, Bandeira F, Lazaretti-Castro M (2014). Recommendations of the Brazilian Society of Endocrinology and Metabology (SBEM) for the diagnosis and treatment of hypovitaminosis D. Arquivos brasileiros de endocrinologia e metabologia.

[11] Rautiainen S, Manson JE, Lichtenstein AH, Sesso HD (2016). Dietary supplements and disease prevention - a global overview. Nat Rev Endocrinol.

[12] Eloi M, Horvath DV, Szejnfeld VL, Ortega JC, Rocha DA, Szejnfeld J, Castro CH (2016). Vitamin D deficiency and seasonal variation over the years in Sao Paulo, Brazil. Osteoporos Int.

[13] Schalka S, Steiner D, Ravelli FN, Steiner T, Terena AC, Marcon CR, Ayres EL, Addor FA, Miot HA, Ponzio H (2014). Brazilian consensus on photoprotection. An Bras Dermatol.

[14] Correa Mde P (2015). Solar ultraviolet radiation: properties, characteristics and amounts observed in Brazil and South America. An Bras Dermatol.

[15] Nabak AC, Johnson RE, Keuler NS, Hansen KE (2014). Can a questionnaire predict vitamin D status in postmenopausal women?. Public Health Nutr.

[16] Summaries for patients (2015). Screening for vitamin D deficiency in adults: US preventive services task force recommendation statement. Ann Intern Med.

[17] Hanwell HE, Vieth R, Cole DE, Scillitani A, Modoni S, Frusciante V, Ritrovato G, Chiodini I, Minisola S, Carnevale V (2010). Sun exposure questionnaire predicts circulating 25-hydroxyvitamin D concentrations in Caucasian hospital workers in southern Italy. J Steroid Biochem Mol Biol.

[18] Fitzpatrick TB (1988). The validity and practicality of sun-reactive skin types I through VI. Arch Dermatol.

[19] Eloi M, Horvath DV, Ortega JC, Prado MS, Andrade LE, Szejnfeld VL, de Moura Castro CH (2017). 25-Hydroxivitamin D serum concentration, not free and bioavailable vitamin D, is associated with disease activity in systemic lupus erythematosus patients. PLoS One.

[20] Sham L, Yeh EA, Magalhaes S, Parra EJ, Gozdzik A, Banwell B, Hanwell HE (2015). Evaluation of fall sun exposure score in predicting vitamin D status in young Canadian adults, and the influence of ancestry. J Photochem Photobiol B.

[21] Maeda SS, Saraiva GL, Hayashi LF, Cendoroglo MS, Ramos LR, Correa Mde P, Henrique de Mesquita C, Lazaretti-Castro M (2013). Seasonal variation in the serum 25-hydroxyvitamin D levels of young and elderly active and inactive adults in Sao Paulo, Brazil: The Sao PAulo Vitamin D Evaluation Study (SPADES). Dermatoendocrinol.

[22] Feizabad E, Hossein-Nezhad A, Maghbooli Z, Ramezani M, Hashemian R, Moattari S (2017). Impact of air pollution on vitamin D deficiency and bone health in adolescents. Arch Osteoporos.

[23] Vimaleswaran KS, Berry DJ, Lu C, Tikkanen E, Pilz S, Hiraki LT, Cooper JD, Dastani Z, Li R, Houston DK (2013). Causal relationship between obesity and vitamin D status: bi-directional Mendelian randomization analysis of multiple cohorts. PLoS Med.

[24] Daly RM, Gagnon C, Lu ZX, Magliano DJ, Dunstan DW, Sikaris KA, Zimmet PZ, Ebeling PR, Shaw JE (2012). Prevalence of vitamin D deficiency and its determinants in Australian adults aged 25 years and older: a national, population-based study. Clin Endocrinol.

[25] Bolek-Berquist J, Elliott ME, Gangnon RE, Gemar D, Engelke J, Lawrence SJ, Hansen KE (2009). Use of a questionnaire to assess vitamin D status in young adults. Public Health Nutr.

