# Supplementary figures and images for: Screening for vitamin D deficiency in a tropical area: results of a sun exposure questionnaire
Source: BMC Endocr Disord. 2018 Jul 3;18:44. doi: 10.1186/s12902-018-0272-0 (PMC6029128; doi:10.1186/s12902-018-0272-0)

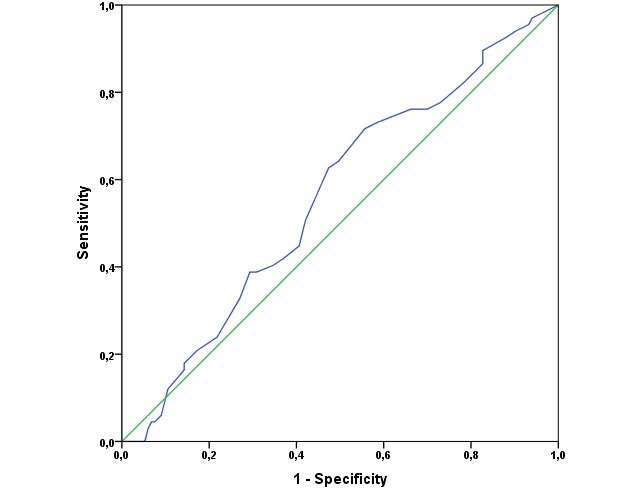

Supplement: Supplementary file 1 — Figure S1. Receiver operating characteristic (ROC) curve showing the performance (sensitivity and specificity) of total sun exposure score to predict vitamin D deficiency (serum 25OHD concentration below 20 ng/mL) in healthy Caucasian individuals living in São Paulo, Brazil. Area under the curve = 0.559; p = 0.172. (DOCX 46 kb) [file 12902_2018_272_MOESM1_ESM.docx]
